# Serotonin Transporter Defect Disturbs Structure and Function of the Auditory Cortex in Mice

**DOI:** 10.3389/fnins.2021.749923

**Published:** 2021-10-06

**Authors:** Wenlu Pan, Jing Pan, Yan Zhao, Hongzheng Zhang, Jie Tang

**Affiliations:** ^1^Department of Physiology, School of Basic Medical Sciences, Southern Medical University, Guangzhou, China; ^2^Functional Nucleic Acid Basic and Clinical Research Center, Department of Physiology, School of Basic Medical Sciences, Changsha Medical College, Changsha, China; ^3^Department of Otolaryngology Head and Neck Surgery, Zhujiang Hospital, Southern Medical University, Guangzhou, China; ^4^Hearing Research Center, Southern Medical University, Guangzhou, China; ^5^Key Laboratory of Mental Health of the Ministry of Education, Southern Medical University, Guangzhou, China

**Keywords:** serotonin transporter, auditory cortex, dendritic spines, tonotopic map, hearing disorder

## Abstract

Serotonin transporter (SERT) modulates the level of 5-HT and significantly affects the activity of serotonergic neurons in the central nervous system. The manipulation of SERT has lasting neurobiological and behavioral consequences, including developmental dysfunction, depression, and anxiety. Auditory disorders have been widely reported as the adverse events of these mental diseases. It is unclear how SERT impacts neuronal connections/interactions and what mechanism(s) may elicit the disruption of normal neural network functions in auditory cortex. In the present study, we report on the neuronal morphology and function of auditory cortex in SERT knockout (KO) mice. We show that the dendritic length of the fourth layer (L-IV) pyramidal neurons and the second-to-third layer (L-II/III) interneurons were reduced in the auditory cortex of the SERT KO mice. The number and density of dendritic spines of these neurons were significantly less than those of wild-type neurons. Also, the frequency-tonotopic organization of primary auditory cortex was disrupted in SERT KO mice. The auditory neurons of SERT KO mice exhibited border frequency tuning with high-intensity thresholds. These findings indicate that SERT plays a key role in development and functional maintenance of auditory cortical neurons. Auditory function should be examined when SERT is selected as a target in the treatment for psychiatric disorders.

## Introduction

Serotonin, one of the most widely spread neurotransmitters in the central nervous system, has been known to play a critical role in brain morphogenesis and functions (Azmitia, 2001; Gaspar et al., 2004). The defects of serotonergic neurons are related to many psychiatric disorders, including depression, anxiety, and autism spectrum disorder (ASD) (Simpson et al., 2011). Acting as a key regulator of serotonergic activity, the serotonin transporter (SERT) is usually selected as the target of antidepressant treatments. SERT represents a potential mediator for anxiety- and depression-related behaviors. However, chronic exposure to selective serotonin reuptake inhibitors (SSRIs) was reported to elicit hearing disorders, such as tinnitus (Kehrle et al., 2015; Pattyn et al., 2016), auditory hallucinations (Kogoj, 2014), and hearing loss (Blazer and Tucci, 2018).

However, how the functions of SERT affect the auditory system remains unclear. Both increases and decreases in serotonin levels during early development have been found to impair the formation and function of the primary somatosensory cortex of rodents (Cases et al., 1996; Persico et al., 2000; Jennings et al., 2006). Pre- and post-natal exposure to the SSRI disturbed the chemoarchitecture of the mouse auditory cortex (AC) and resulted in ASD-like behavior (Simpson et al., 2011). Our previous study found that both SSRI treatment and SERT knockout (KO) did not change the auditory brain responses but abolished the auditory mismatch negativity in adult animals (Pan et al., 2020). These investigations suggest that SERT may affect the auditory functions by manipulating the serotonin level and serotonergic neurons of AC.

In rodents, 2 weeks after birth, layer IV afferent neurons in the primary visual, auditory, and somatosensory cortex are innervated by aggregates of serotonin-containing processes (D’Amato et al., 1987; Blue et al., 1991). At present, the morphological and especially the functional implications of SERT in AC remain unclear. In the present study, we examined the fine dendritic structure of the neurons in primary AC. We found that although expression of SERT is low in the AC, both pyramidal neurons and interneurons in SERT KO mice showed significant reductions in dendritic length, the number, and density of dendritic spines than did wild-type (WT) mice. The electrophysiological features of AC were also impaired by the SERT deficit. These results suggest that SERT plays a key role in development and functional maintenance of AC. This may explain the observed hearing disorders in patients utilizing drugs that target SERT, such as SSRIs, in the treatment for psychiatric disorders.

## Materials and Methods

All the animal experiments involved in this study were approved by the Institutional Animal Care and Use Committee (IACUC) of Southern Medical University. The principles formulated by the Animal Care Committee of Southern Medical University were followed throughout the experiment.

### Subjects

SERT KO mice were derived from the Jackson Laboratory (Stock No. 008,355) and backcrossed with C57BL/6J background mice (Bengel et al., 1998). Polymerase chain reaction (PCR) protocol used for genotyping SERT KO mice has been reported in our previous literature (Pan et al., 2020). Male and female mice aged 2–4 months and weighting 20–26 g were employed in our test. Animals were housed in a room maintained at 22°C (±2°C) and kept on a 12:12 light/dark cycle with lights on at 8:00 a.m.

### Immunohistochemistry

In this study, adult C57BL/6J mice were deeply sedated with pentobarbital sodium (50 mg/kg) administered *via* intraperitoneal injection. The mice were perfused intravascularly *via* the left ventricle with phosphate-buffered saline (PBS; pH 7.4), followed by a fixative, viz., 4% (w/v) paraformaldehyde. The mice were decapitated; the brain and cochleae were harvested and fully fixed in 4% paraformaldehyde, at 4°C overnight. Then the brain was sectioned on a freezing microtome to a thickness of 50 μm. The cochleae were washed in PBS, then placed into 10% ethylenediaminetetraacetic acid (EDTA) decalcifying solution, and replaced with fresh decalcifying solution every day for 6–8 days. Decalcification is terminated when the cochleae were transparent and elastic. Then the cochleae were sliced up parallel to the modiolar plane of the cochlea, and thickness of the slices was 10 μm. Brain and cochleae tissue samples were permeabilized in 0.3% Triton X-100 (Gibco, Grand Island, NY, United States) for 1 h and immunoblocked with a solution of 10% goat serum albumin for an additional hour. The specimens were incubated overnight at 4°C with SERT antibody (Millipore, Billerica, MA, United States; cat. no. 2828614) diluted in 10% goat serum albumin. After several washes in PBS, the specimens were then incubated with the Alexa-Fluor-488-conjugated secondary antibody at a concentration of 1:1,000 for 1 h at room temperature. To assign neurons, sections were counterstained for 20 min at room temperature with a fluorescent dye NeuroTrace 530/615 (1:100, Invitrogen, Carlsbad, CA, United States). Samples were then washed with PBS for three times and examined by using a Nikon confocal microscope (Nikon Instruments Inc., Melville, NY, United States).

### Golgi Staining

Golgi staining was used to visualize the dendritic branching complexity and spines of the neurons in the mice. Mice were anesthetized with 10% chloral hydrate. The brains were taken after being fully infused with 0.9% saline and 4% paraformaldehyde, successively. Then, the brains were immersed in Golgi–Cox solution (Glaser and Loos, 1981) and stored at room temperature for 2 weeks in the dark. Next, the brains were transferred to a 30% sucrose solution and dehydrated at 4°C for 2–5 days, avoiding light. The brain tissues were completely coated with OCT embedding agent (Tissue-Tek 4583; Sakura Finetek United States, Inc., Torrance, CA, United States). The 100-μm-thick sections were prepared on gelatin-coated slides in a coronal plane parallel to the base and left to air-dry away from light for 2 days before being processed for Golgi–Cox impregnation. The brain slides were put into a special opaque staining box, and Golgi–Cox staining was performed as the literature (Zhang et al., 2009).

### Sholl’s Analysis

Dendrites in each of the selected neurons were quantitatively analyzed using Sholl’s concentric circle method (Sholl, 1953). The neurons were selected from layers IV and II/III in AC using the following criteria: (1) the cell body was in the subregion of interest, (2) the staining of the branches was efficient and complete throughout the length, and (3) the branches were isolated from their neighbors. A series of concentric rings, spaced 20 μm apart, were placed over the neuron and centered on the cell body, and the number of dendrites intersecting each circle in the series of concentric circles was counted blind to the experimental conditions to estimate the total dendritic length, branch points, and dendritic complexity.

### Dendritic Spine Density Analysis

Spine analyses were conducted blind to the experimental conditions on coded Golgi impregnated brain sections containing the AC. Spines were examined on dendrites of pyramidal neurons and inter neurons. Briefly, all protruding dendritic spines were counted on per 25-μm dendritic segments. Spine density was expressed as the number of spines per 25 μm. Two to three dendritic segments were analyzed per neuron. Only intact, properly stained, and unbranched dendritic segments were included in the analyses. To acquire images for spine analysis, the dendritic segments were imaged under bright field illumination on a Zeiss Axioimager microscope (Carl Zeiss, Oberkochen, Germany) with a 63× oil immersion objective.

### Recording in the Primary Auditory Cortex (AI)

WT and SERT KO mice weighing 20–26 g were anesthetized by pentobarbital sodium (30 mg/kg, i.p.), followed by atropine sulfate (0.25 mg/kg, i.h.) to prevent asphyxia. The level of anesthesia was maintained by additional dosages of sodium pentobarbital (30 mg/kg, i.p.) administered approximately every 40 min throughout the physiological experiments. Under anesthesia, the mouse’s head was fixed in a head holder by rigidly clamping on a nail about 1.5 cm long fixed on the surface of the skull vertically with dental cement (Tang et al., 2008; Tang and Suga, 2009).

The scalp, muscles, and soft tissues of the skull were then removed; an opening above the left AC was made using a dental driller; and the dura was gently removed. The anatomical location of AI in AC was marked according to the brain map (bregma −2.7 mm, left/right of the midline 3.5 mm) (Umbricht et al., 2005), and the size of the parietal open window was 0.2 × 0.2 mm^2^. The cortex was maintained under artificial cerebrospinal fluid to prevent desiccation. The mouse was placed on a feedback-controlled heating pad to maintain its body temperature at 37°C. All electrophysiological experiments were performed in a soundproof and echo-attenuated chamber.

Microelectrodes with a ∼1-μm tip diameter (7–12 MΩ, filled with 3 mol/L of KCl) were used for recordings. At every recording site, the microelectrode was lowered orthogonally into the cortex to depths of 200–375 μm (layers II/III) or 475–600 μm (layers IV/V), where the evoked spikes of a neuron or a small cluster of neurons were collected. After the best frequency (BF) of neurons was found at a recording point once, the electrode was moved toward the rostral or caudal side to the next point 200–300 μm away from the previous recording point, and the same measurement was repeated. Complete examinations were repeated in this way until no response at the two adjacent voice-induced recording sites was observed in any direction.

Pure tones were generated and played with a TDT 3 system (Tucker-Davis Technologies, Alachua, FL, United States) for auditory mapping. A real-time processor (RP2.1) and a program written in RPvdsEx software were used to synthesize the sound signals. Sound intensity was adjusted by an attenuator (PA5). The synthesized sound signal was amplified by an electric driver (ED1) with an open-field speaker (ES1). Before the OpenEx software (sampling rate = 25 kHz) recording, the speakers were calibrated with 1/8- and 1/4-inch microphones (Brüel and Kjaer 4138, 4135, Naerum, Denmark). Neural signals were amplified 10,000× using a digital amplifier (RA16) with a 0.3- to 3-kHz filter and monitored online by a software Brainware (Tucker-Davis Technologies). Frequency–intensity receptive fields (RFs) were reconstructed in detail by presenting pure tones (50-ms duration, 5-ms ramps) of six frequencies (2–32 kHz) at nine sound intensities [0–90 dB sound pressure level (SPL), in 10-dB increments] at a rate of two stimuli per second. The tones were presented in a random, interleaved sequence.

To generate the cortical map, we used Matlab functions to create colored polygons, constructed by connecting a record point and intermediate points between four and six adjacent record points (Bao et al., 2003). The electrode penetration point was located in its center, and each polygon was the BF with responsive neurons at this point. In the topological diagram, the RF of auditory neurons in area AI was continuous, single-peaked, and V-shaped. Moreover, the BF of the neurons in this region was tonotopically organized, with high frequencies in the rostrally and low frequencies in the caudally.

### Data Processing and Statistical Analysis

We defined the minimum threshold (MT) and BF to enable neurons to respond to the minimum stimulus sound intensity at the corresponding sound frequency when firing maximum number of spikes at this site. The BF of the cortical region AI was defined as the frequency at the tip of the tuning curve, that is, the sound frequency corresponding to the MT of the neuron at this place. The sharpness of the frequency-tuning curve, defined for all recording sites, was represented by the value Q30 (Tang et al., 2012). Q30 was equal to the BF divided by the bandwidth value of 30 dB above the MT within the frequency–intensity RF range. The larger the Q30, the sharper the tuning curve representing the neurons, and the better the frequency selectivity of the neurons. A customized MatLab program (MathWorks, Natick, MA, United States) was used to analyze and plot tonotopic map of A1. Tonotopic index (TI) was used to evaluate the tonotopic organization by following the methods described in previous studies (Zhang et al., 2001; Bao et al., 2004).

We used SPSS 20 software (IBM, Armonk, NY, United States) to perform statistical analysis. The two-tailed *t*-tests (for unpaired comparisons) and two-way ANOVA (for multiple-group comparisons) were used to test for significant differences between groups. Statistical significance was defined as *p* < 0.05. GraphPad Prism 7 (GraphPad Software, San Diego, CA, United States) was used for plotting.

## Results

**FIGURE 1:**
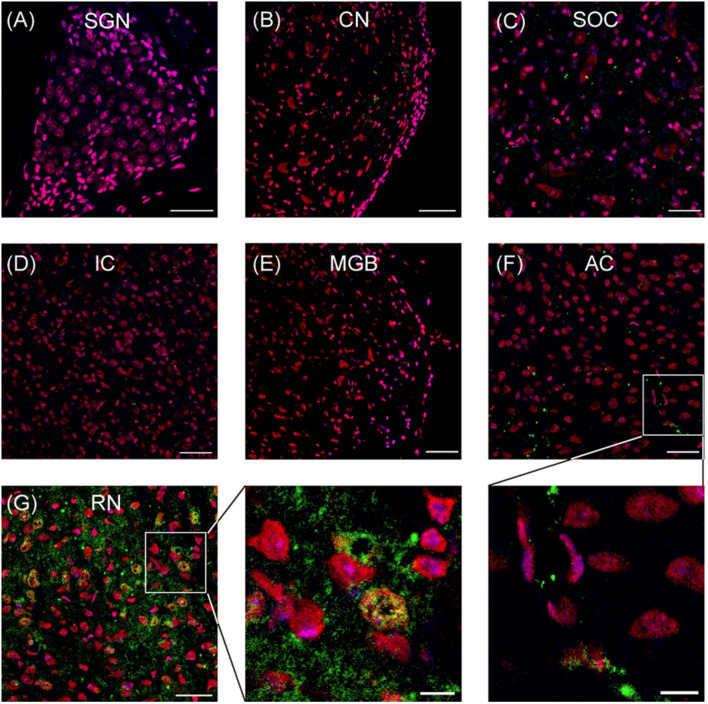
Representative images show the serotonin transporter (SERT) expression in different regions of auditory system. **(A–G)** Confocal images show the SERT (green) expression in the spiral ganglion neurons (SGN), cochlear nuclei (CN), superior olivary complex (SOC), inferior colliculus (IC), medial geniculate body (MGB), auditory cortex (AC), and raphe nuclear complex (RN). Insets are the magnified images of the boxes in **(F,G)**. The soma and nucleus of neurons are colored in red (Nissl) and blue (DAPI), respectively. SERT is colored in green. Scale bars: 50 μm for **(A–G)** and 10 μm for insets.

By using immunostaining, the expression of SERT in the auditory neural system was examined systematically. As shown in Figure 1, no robust SERT expression was detected in the cochlea (spiral ganglion neurons; Figure 1A), auditory brainstem (cochlear nuclei, superior olivary complex, and inferior colliculus; Figures 1B–D), or auditory thalamus (medial geniculate body; Figure 1E). Hence, we examined the SERT expression in the primary AC. Our data from AC exhibit sporadic spread of SERT immunoreactive puncta, which represents the SERT immunoreactive fibers (Figure 1F). However, the SERT immunoreactive puncta was usually detected around the soma of cortical neurons (inset of Figure 1F) with a much lower density than the expression in the raphe nuclear complex (Figure 1G). This result suggests that AC neurons receive sparse serotonergic projections, if any.

**FIGURE 2:**
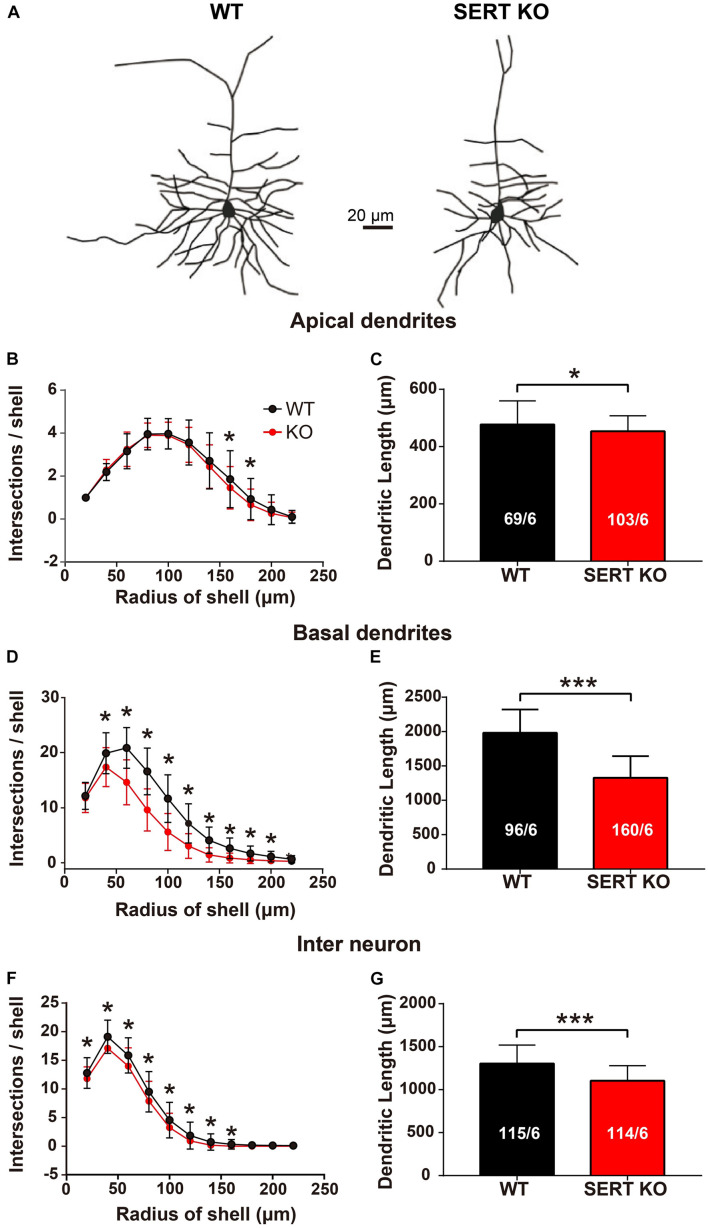
Serotonin transporter (SERT) knockout (KO) degrades the dendrites of layer IV pyramidal neurons and layer II/III interneurons in auditory cortex. **(A)** Representative dendritic morphology of layer IV pyramidal cells in auditory cortex from wild-type (WT) and SERT KO mice. Scale bar: 20 μm. **(B,C)** Comparing the number of intersections and the total length of the apical dendrites of layer IV pyramidal cells in WT mice with SERT KO mice. **(D,E)** Comparing the number of intersections and the total length of the basal dendrites of layer IV pyramidal cells in WT mice with SERT KO mice. **(F,G)** The number of intersections and the total length of the dendrites of layer II/III interneurons were significantly reduced in SERT KO mice, compared with WT mice. The number of intersections of dendrites was measured with 20-μm concentric spheres centered on the soma by Sholl’s analysis. The numbers in the column indicate the numbers of neurons/numbers of animals analyzed. Data are presented as means ± SD. * *p* < 0.05; *** *p* < 0.001, Student’s *t*-tests and two-way ANOVA.

To determine the roles of these serotonergic projections, the morphology of AC neurons from SERT KO mice was examined in comparison with that of WT mice. Our data show that for pyramidal cells in layer IV, the number of intersections and the total length of dendrites were significantly reduced in both apical and basal dendrites of the SERT KO AC neurons (Figure 2). Moreover, the reduction of dendrites was also found in layer II/III interneurons of SERT KO AC (Figures 2F,G).

**FIGURE 3:**
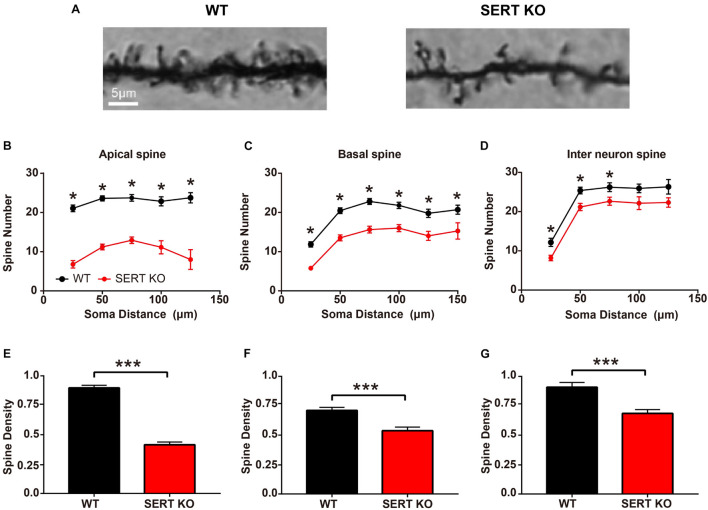
Dendritic spines density decreased in serotonin transporter (SERT) knockout (KO) mice. **(A)** Representative photomicrographs of dendritic spines of basal dendrites in layer IV pyramidal neurons from wild-type (WT) and SERT KO mice. **(B,C)** Comparing the total number of apical and basal dendritic spines at a distance of per 25 μm from the cell body of layer IV pyramidal cells in WT mice with SERT KO mice, respectively. **(D)** Comparing the total number of dendritic spines at a distance of per 25 μm from the cell body of layer II/III interneurons in WT mice with SERT KO mice. **(E–G)** The dendritic spine densities of apical and basal dendrites in layer IV pyramidal neurons and in layer II/III interneurons of the auditory cortex. Data are presented as means ± SD. *n* = 6 for each group. * *p* < 0.05, *** *p* < 0.001, Student’s *t*-test and two-way ANOVA. Scale bar: 5 μm.

Most excitatory synaptic connections occur on dendritic spines. Spines can individually detect the temporal coincidence of pre- and post-synaptic activities and thus serve as basic functional units of neuronal integration (Gray, 1959; Yuste and Denk, 1995; Spacek and Harris, 1998; Arellano et al., 2007). To further determine whether synaptic function at neuronal junctions was altered in SERT KO mice, we looked at the morphologic structure of the dendritic spines of AC neurons. We measured the number and density of total dendritic spines in layer IV pyramidal cells and layer II/III interneurons from the AC of WT and SERT KO mice (Figure 3A). In apical dendrites, the total number of dendritic spines of layer IV pyramidal cells was less in SERT KO mice [vs. WT, *F*(1,159) = 252.5, *p* < 0.0001, two-way ANOVA]. Significant reduction was observed at a distance of 25–125 μm from cell bodies (vs. WT, *p* < 0.001, two-tailed *t*-test; Figure 3B). The mean density of apical dendritic spines reduced significantly in SERT KO mice (vs. WT, *p* < 0.0001, two-tailed *t*-test; Figure 3E). The total number of basal dendritic spines of layer IV pyramidal cells was also reduced in SERT KO mice [vs. WT, *F*(1,356) = 109.6, *p* < 0.0001, two-way ANOVA] and significantly reduced at a distance of 25–150 μm from cell bodies (vs. WT, *p* < 0.001, two-tailed *t*-test; Figure 3C). Meanwhile, the mean density of basal dendritic spines reduced significantly in SERT KO mice (vs. WT, *p* < 0.0001, two-tailed *t*-test; Figure 3F). However, the total number of dendritic spines in layer II/III interneurons in SERT KO mice was not significantly different [vs. WT, *F*(1,292) = 10.93, *p* = 0.011, two-way ANOVA]. The density was only found reduced at a distance of 25–75 μm from cell bodies (vs. WT, *p* < 0.05, two-tailed *t*-tests; Figure 3D). The mean density of dendritic spines was also significantly reduced in SERT KO mice (vs. WT, *p* < 0.0001, two-tailed *t*-test; Figure 3G). Together with the results of our Sholl’s analysis, these morphological data implied that synaptic transmission between the subcortical and cortical neurons, as well as the neurons within the AC, might be weakened in the auditory system of SERT KO mice. These defects may affect the functions of SERT KO AC neurons.

Electrophysiological experiments were conducted to investigate the functional alteration of auditory neurons in primary AC (AI) of SERT KO mice. Previous studies have found that neurons at different locations within the same layer of AI respond to different frequencies (Kelly and Sally, 1988; Kaas et al., 1999; Zhang et al., 2001; de Villers-Sidani et al., 2007). Therefore, we measured the “frequency-tonotopic map” of neurons in the AI region of cortex. A total 246 sites and 243 sites were recorded from the auditory cortical area of WT and SERT KO mice, respectively. Normally in WT mice, high-frequency sensitive neurons are located in the rostral sites of the AI region, and low-frequency sensitive neurons are located in caudal side (Stiebler et al., 1997).

**FIGURE 4:**
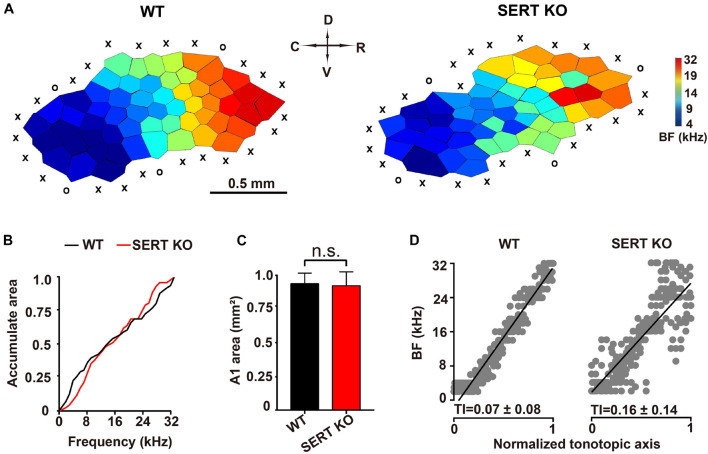
Disturbed tonotopic organization of primary auditory cortex in serotonin transporter (SERT) knockout (KO) mice. **(A)** Representative A1 characteristic-frequency map from a wild-type (WT) mouse (left) and a SERT KO mouse (right). Scale bar: 0.5 mm. X, unresponsive cortical site; O, non-A1 cortical site (see section “Materials and Methods”). **(B)** Plotting accumulated response areas at best frequency for neurons of primary auditory cortex in WT and SERT KO mice. **(C)** The total areas of primary auditory cortex in WT mice and SERT KO mice. **(D)** The best frequencies of all recorded sites in WT and SERT KO mice were plotted against a normalized tonotopic axis. The tonotopic index (TI) represents the increased scatter of best frequencies around the ideal tonotopic axis (black diagonal line) (see section “Materials and Methods”). Data are presented as means ± SD. ns, no statistical difference, *p* > 0.05. Student’s *t*-test.

As shown in the representative data in Figure 4A, the BFs of AC neurons were distributed regularly from the rostral to caudal sites, forming a compact and ordered “tonotopic map” in WT mice. However, for AC neurons of SERT KO mice, their frequency selectivity did not show a systematic organization, although the neurons that respond to high frequencies were still mostly located to the rostral side. We further analyzed the frequency range and the total area of the AI region. No statistical difference was found between SERT KO and WT mice (*p* > 0.05, two-tailed *t*-test; Figures 4B,C). The BFs of all recorded sites were plotted against a normalized AC axis (Figure 4D). The distribution of BFs was quantified with the TI, which was significantly increased in SERT KO mice (*p* < 0.01, two-tailed *t*-test). These results showed that a disrupted tonotopic map was found in the AC of SERT KO mice, suggesting that SERT may mainly affect the neuron’s tuning property.

**FIGURE 5:**
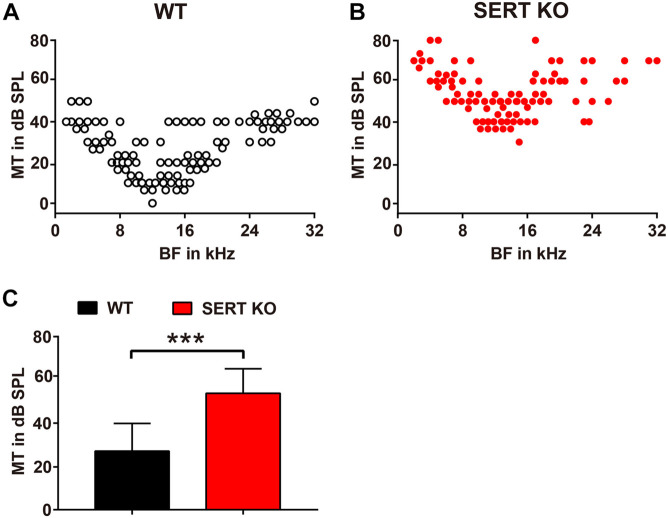
Serotonin transporter (SERT) knockout (KO) elevates the threshold of neurons in primary auditory cortex. Pooled data show minimum thresholds at the best frequency of auditory neurons recorded from the A1 region of wild-type (WT) **(A)** and SERT KO mice **(B)**. WT: *n* = 6, recording site = 246. SERT KO: *n* = 6, recording site = 243. **(C)** The average minimum threshold (MT) of neurons in SERT KO mice was significantly higher than that in WT mice. Data are means ± SD. *** *p* < 0.001. Student’s *t*-test.

We then measured the BF and the MT of AC neurons recorded from SERT KO and WT mice, by which the frequency selectivity and sound sensitivity of neurons were compared. The BFs and MTs of SERT KO and WT neurons were pooled in Figures 5A,B, respectively. Interestingly, although no change was found in BFs, the MTs of SERT KO neurons were significantly elevated. The MTs of WT neurons were generally below 40 dB SPL with an average of 27.09 ± 12.81 dB SPL. However, the MTs of SERT KO neurons were significantly higher with an average of 53.85 ± 11.33 dB SPL (*p* < 0.0001, two-tailed *t*-test; Figure 5C). These data suggest that individual neurons in SERT KO mice were significantly less sensitive to sound stimulation.

**FIGURE 6:**
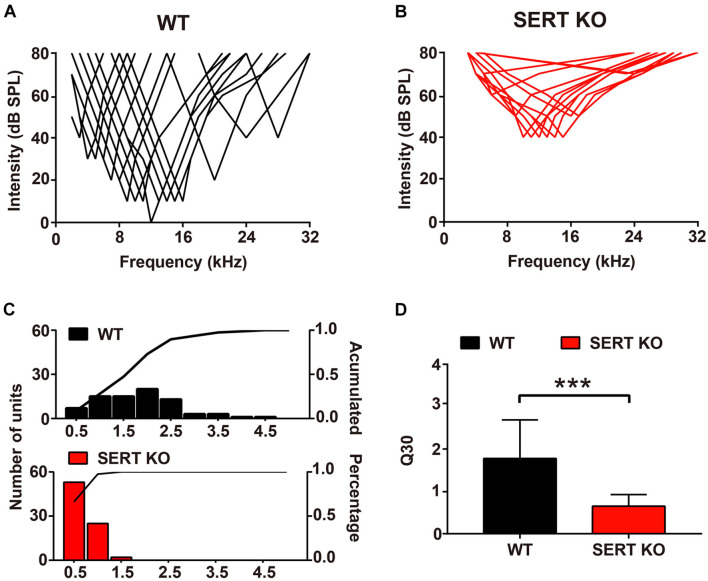
Broader frequency tuning for primary auditory cortical neurons in serotonin transporter (SERT) knockout (KO) mice. Pooled data show the frequency-tuning curves of cortical neurons in wild-type (WT) **(A)** and SERT KO **(B)** AI. **(C)** The distribution of the Q30 values for cortical neurons in WT (top panel) and SERT KO (bottom panel) AI. The lines in the panels represent the cumulative percentage of the Q30 value. **(D)** The mean Q30 value of all auditory neurons recorded from WT mice and SERT KO AI. Data are presented as means ± SD. *n* = 6 for each group. *** *p* < 0.001. Student’s *t*-test.

Auditory neurons respond not only to their most sensitive frequency (i.e., BF) but also to other frequencies of sound. One of the most important functions of auditory neurons is that they can selectively respond to sound within a range of frequencies. In WT mice, individual neurons respond to sound frequencies in addition to the BF at sound levels above MT. These frequency-threshold intensity points formed a “V-shaped” frequency-tuning curve (Figure 6A). In SERT KO mice, the frequency-tuning curves of cortical neurons have much wider bandwidth (Figure 6B). To evaluate the frequency selectivity of a single neuron, we used “Q30” value to analyze the changes in frequency selectivity of AC neurons. The higher the value of Q30, the sharper the frequency-tuning curve of the neuron, and the better the frequency selectivity. For AC neurons of WT mice, their Q30 values mostly ranged from 1 to 2.5, with an average of 1.78 ± 0.91 (Figures 6C,D). But in SERT KO mice, Q30 values of most AC neurons were below 1 (0.65 ± 0.27, mean ± SD; Figures 6C,D). This result suggested that AC neurons in SERT KO mice have less selectivity to sound frequency.

## Discussion

Our results of immunohistochemical study in the whole auditory system showed that SERT was rarely expressed in the auditory neurons, including those in the AC, of adult WT mice (Figure 1). These data suggest that the morphological and functional defects observed in AC neurons of SERT KO mice may not due to the disruption of serotonergic innervations directly but more likely related to the effects of SERT deficit on cortical circuit development.

As the staining results showed, the length of basal dendrites of pyramidal cells was significantly shortened (Figure 2), and the number and density of dendritic spines were significantly reduced in SERT KO mice (Figure 3), suggesting that the ability of pyramidal cells to receive input information was decreased, which might cause less sensitivity to sound response (Figure 5). Moreover, the reduction of the length, the number, and density of dendritic spines in inhibitory interneurons of SERT KO mice suggests that the reduced inhibitory of the interneurons might cause less frequency selectivity of neurons (Figure 6). However, deficient SERT may change the neural morphology in different ways in different brain regions. The length of dendrite and the density of dendritic spines were increased in the infralimbic cortex of SERT KO mice. Meanwhile, SERT KO has little effects on the morphology of basolateral amygdala neurons (Wellman et al., 2007). These different results suggest that the morphology of neurons was also specifically determined by their circuits; even the SERT was abolished in the early stage of the development. Interestingly, many evidences have suggested that SERT is expressed not only on serotonin neurons but also on other neurons such glutamatergic neurons during development. The loss of SERT in these neurons may affect the neural circuit in the cortex during development (Chen et al., 2015). This may explain why the morphology of AC neurons was altered by SERT KO, although few direct serotonergic projections were detected in AC of WT animals.

SERT is a target of SSRIs treatments and regulates the activity of serotonergic system (Kalueff et al., 2009). In the present study, we examined the role of SERT function in primary AC by using SERT KO mice. In WT mice, the primary AC formed a compact and ordered “tonotopic map”; the optimal response frequencies from the rostral to caudal side was regularly distributed. But in SERT KO mice, the primary AC formed a distorted tonotopic organization (Figure 4). However, in SERT KO mice, the disordered frequency topology did not show a systematic decrease in frequency, although the neurons that respond to high frequencies were still mostly medial to the rostral side, and the neurons that respond to low frequencies were still mostly lateral to the caudal. Next, we further analyzed the total area of the tonotopic map in the AI region and the frequency range of the response to sound (Figure 4B). There was no statistical difference between SERT KO mice and WT mice (Figure 4B). It suggests that SERT may mainly affect the orderliness of the neuron’s frequency response. Similarly, manipulations of rodent brain serotonin levels during early development, through either increases (produced in SERT or monoamine oxidase KO mice) or decreases (produced by parachlorophenylalanine or other treatments), alter the formation of the whisker (barrel) representation in the primary somatosensory cortex and promote aggressive and/or anxiety-related behaviors (Cases et al., 1995, 1996; Persico et al., 2000; Persico et al., 2001; Holmes et al., 2003; Jennings et al., 2006). In our previous study, auditory mismatch negativity response was found abolished by SERT KO, indicating the low ability in frequency discrimination (Pan et al., 2020). This finding was supported by the functional changes of AC observed in the present study. Under the administration of SSRIs, the animal showed anxiety-related and ASD-like behavior (Simpson et al., 2011; Pan et al., 2020). Although the abnormal behavior and auditory function were quite consistent in SERT KO and SSRI-administered animals, the direct evidence is still very limited for us to understand the effects of SSRIs on development and function of cortical circuits. However, the SERT KO mouse model used in the present study is constitutive and global. To investigate the role of SERT and SSRI in the development of ASD and anxiety, a knock-in SERT mouse model, such as SERT Ala56, should be expected in future studies (Siemann et al., 2017).

One of previous findings related to serotonin and early cortical organization found that the primary sensory cortex of mice, especially the layer IV neurons of visual, auditory, and somatosensory cortex, is transiently innervated with dense 5-HT-containing projection (D’Amato et al., 1987; Blue et al., 1991; Bennett-Clarke et al., 1994). Moreover, in early brain development, 5-HT is taken up into glutamatergic thalamocortical terminals (Bennett-Clarke et al., 1996; Lebrand, 1996) and used in combination with the 5-HT 1B receptor on layer IV afferents (Salichon et al., 2001). Serotonin plays a trophic role in brain morphogenesis, including cell proliferation, migration, and differentiation. It is also one of the first neurotransmitters to appear in the central nervous system (Azmitia, 2001; Gaspar et al., 2004). We found that the typical dendritic morphology was shortened (Figure 2) and that the density of dendritic spines was lower in SERT KO mice compared with WT mice (Figure 3). In SERT KO mice, the deficit of SERT may alter neural morphology and distort tonotopic organization in primary AC.

The structure and arborization of dendrites have a profound impact on the processing of neuronal information because they determine the extent of a neuron’s synaptic field. Thousands of spines stud the pyramidal cell’s apical and basal dendritic branches, increasing the neuron’s receptive surface area and allowing for integration of thousands of excitatory signals to influence the output (Spruston, 2008). Obviously, dendritic spines receive most of the excitatory impulses of a pyramidal cell, consistent with the information processing capacity of the neuron. A cell with spiny processes in homologous nuclei has more spines, the higher level of the subject in the animal series. Thus, as an example in vertebrates, the Purkinje cell of birds shows fewer spines than that of mammals.

Since neural circuits are defined by inter-neuronal communications, output precision in individual cells becomes essential to network function. Cortical interneurons use rhythmic inhibition to create narrow windows for effective excitation, entraining excitatory pyramidal cells to fire certain oscillatory patterns (Beierlein et al., 2000; Uhlhaas and Singer, 2010). Moreover, optogenetic manipulation of specific excitatory and inhibitory circuits directly caused changes in social and cognitive behaviors in mice (Yizhar et al., 2011). Thus, circuits tend to be malleable but vulnerable during post-natal development. Notably, several neurodevelopmental disorders, such as ASD and schizophrenia (SCZ), manifest during this plasticity period (Zoghbi, 2003; Hossein and Folsom, 2009).

Interestingly, in our results, the neuron functions altered; especially, the MT for a BF of SERT KO mice was significantly higher than 40 dB (Figure 5). It suggested that absent SERT reduced the neurons’ intensity sensitivity to response. Moreover, in SERT KO mice, Q30 values were mostly below 1 (Figure 6). Meanwhile, the frequency-tuning curve was dull, and the frequency selectivity of neurons was significantly worse (Figure 6). It suggested that neurons in SERT KO mice showed less frequency selectivity to response.

All of these results may be related to shortened typical dendritic morphology of neurons (Figure 2) and the lower density of dendritic spines of primary AC in SERT KO mice (Figure 3), which may result in weakened synaptic information transmission between the cortical pyramidal cells and the interneurons of AC in SERT KO mice. The altered function of the primary AC suggests that SERT plays a critical role in circuit development and function.

## Data Availability Statement

The original contributions presented in the study are included in the article/supplementary material, further inquiries can be directed to the corresponding authors.

## Ethics Statement

The animal study was reviewed and approved by Institutional Animal Care and Use Committee (IACUC) of Southern Medical University.

## Author Contributions

HZ and JT designed the experiments. WP, JP, and YZ performed the experiments. All authors wrote, reviewed, edited, and approved the final manuscript.

## Conflict of Interest

The authors declare that the research was conducted in the absence of any commercial or financial relationships that could be construed as a potential conflict of interest.

## Publisher’s Note

All claims expressed in this article are solely those of the authors and do not necessarily represent those of their affiliated organizations, or those of the publisher, the editors and the reviewers. Any product that may be evaluated in this article, or claim that may be made by its manufacturer, is not guaranteed or endorsed by the publisher.
